# The effects of flavonoids, green tea polyphenols and coffee on DMBA induced LINE-1 DNA hypomethylation

**DOI:** 10.1371/journal.pone.0250157

**Published:** 2021-04-20

**Authors:** Laszlo Szabo, Richard Molnar, Andras Tomesz, Arpad Deutsch, Richard Darago, Ghodratollah Nowrasteh, Timea Varjas, Balazs Nemeth, Ferenc Budan, Istvan Kiss

**Affiliations:** 1 Department of Public Health Medicine, Medical School, University of Pécs, Pécs, Hungary; 2 Institute of Physiology, Medical School, University of Pécs, Pécs, Hungary; 3 Institute of Environmental Engineering, Faculty of Engineering, University of Pannonia, Veszprém, Hungary; 4 Szentagothai Research Centre, University of Pécs, Pécs, Hungary; Semmelweis University, HUNGARY

## Abstract

The intake of carcinogenic and chemopreventive compounds are important nutritional factors related to the development of malignant tumorous diseases. Repetitive long interspersed element-1 (LINE-1) DNA methylation pattern plays a key role in both carcinogenesis and chemoprevention. In our present in vivo animal model, we examined LINE-1 DNA methylation pattern as potential biomarker in the liver, spleen and kidney of mice consuming green tea (*Camellia sinensis*) extract (catechins 80%), a chinese bayberry (*Morella rubra*) extract (myricetin 80%), a flavonoid extract (with added resveratrol) and coffee (*Coffee arabica*) extract. In the organs examined, carcinogen 7,12-dimethylbenz(a)anthracene (DMBA)-induced hypomethylation was prevented by all test materials except chinese bayberry extract in the kidneys. Moreover, the flavonoid extract caused significant hypermethylation in the liver compared to untreated controls and to other test materials. The tested chemopreventive substances have antioxidant, anti-inflammatory properties and regulate molecular biological signaling pathways. They increase glutathione levels, induce antioxidant enzymes, which decrease free radical damage caused by DMBA, and ultimately, they are able to increase the activity of DNA methyltransferase enzymes. Furthermore, flavonoids in the liver may inhibit the procarcinogen to carcinogen activation of DMBA through the inhibition of CYP1A1 enzyme. At the same time, paradoxically, myricetin can act as a prooxidant as a result of free radical damage, which can explain that it did not prevent hypomethylation in the kidneys. Our results demonstrated that LINE-1 DNA methylation pattern is a useful potential biomarker for detecting and monitoring carcinogenic and chemopreventive effects of dietary compounds.

## Introduction

In 2018 the number of newly discovered malignant cancer cases worldwide was estimated to be 18.1 million [1]. Theoretically, 40% of these could be prevented, as mostly environmental effects and lifestyle habits are responsible for carcinogenesis [2]. A significant part of these factors is represented by unhealthy nutrition, oxidative stress, the presence of carcinogenic pollutants and further lifestyle-related factors–which become manifest even at the epigenetic level [3].

However, protective substances–e.g. anti-inflammatory, proapoptotic, antioxidant and cell proliferation inhibitory agents–can also be found among nutritional factors. Indeed, nutritional factors, supported by numerous in vitro, in vivo, and clinical studies, are the most significant ones among carcinoprotective strategies [4–6]. Studies on the antitumor effects of flavonoids, resveratrol and green tea (*Camellia sinensis*) extracts occupy a prominent place among them. (Approximately 600,000 tons of green tea (*C*. *sinensis*) and 131,050 tons of resveratrol is consumed every year in the world.) In particular, antioxidant and anti-inflammatory effects or other molecular biological pathways (such as inhibition of cell proliferation, supporting mismatch repair, proapoptotic effects, inhibition of angiogenesis or promotion of cell differentiation, etc.) can be found in the background of chemopreventive properties of polyphenols e.g. flavonoids, and other food items, e.g coffee (*Coffee arabica*) [4–7]. In 2019/20, worldwide (*C*. *arabica*) production was 93.83 million 60 kilograms bags. When consuming more than four cups of coffee per day, the relative risk (RR) of malignant liver cancer was 0.50 (95% CI: 0.42–0.59) and the RR of colorectal carcinoma was 0.83 (95% CI: 0.75–0.92) [7]. Chinese bayberry (*Morella rubra*) is a subtropical evergreen tree cultivated in southern China and its annual production has reached 1.1 million tons [8]. Chinese bayberry (*M*. *rubra*) has a great potential as antitumor agent based upon in vitro studies using chemotherapy-resistant OVCAR-3 spheroid cells [9]. (For the list of abbreviations see S1 Data.)

In the light of this, it is necessary to continuously monitor the aggregation of harmful and protective environmental effects with potential impact on human health. The study of the mentioned carcinogenic and chemopreventive mechanisms is essential for the development of novel chemopreventive strategies [10] and this is possible only through the use of appropriate predictive biomarkers.

Methylation of DNA is a key factor of carcinogenic and antitumor effects as well as a representative molecular epidemiological biomarker of them. Thus, methylation pattern as a biomarker reflects both the nutritional factors and the mentioned adverse environmental effects. Furthermore, the methylation pattern should also be mentioned as a factor affecting gene expression. The increase in global DNA methylation is carcinoprotective by inhibiting transcription factors (e.g. *C-MYC*, *BCL-2*), leading to slowing down DNA replication [11], or by supporting mechanisms leading to cell cycle arrest [12]. However, global hypomethylation of DNA can reduce the stability of the genome and may lead to the development of several diseases [13].

DNA methylation patterns are generated by DNA methyltransferase (DNMT) enzymes which are able to methylate pyrimidine bases, among them especially cytosine bases (in eukaryotes on cytosine-C5) [14, 15]. DNA methylation occurs in 99.98% at the so-called CpG site (i.e., cytosine preceding guanosine site), which is characterized by a cytosine followed by a guanine base [16]. Its transcriptional regulatory significance is manifested on the tandem repeated CpG sites, on the so-called “CpG islands”. In practice, hypomethylation of oncogenes and hypermethylation of tumor suppressor genes promote tumorigenesis, while the same is true vice versa. Transposons are gene segments that can be translocated by transposase enzymes. Global hypomethylation of the genome enhances the ability of transposons to translocate—which may cause mutations when accidentally inserted into the genome and it may lead to genetic instability, as well as to carcinogenesis [15]. An illustrative example of this is an animal experiment in which the transposase enzyme system, called „Sleeping Beauty”, artificially inserted into the genome, mimicking the action of the carcinogen 7,12-dimethylbenz(a)anthracene (DMBA) as well, specifically activates oncogenes (e.g. *HA-RAS* proto-oncogene) [17]. The repetitive long interspersed element-1 (LINE-1) contains about 6,000 base pairs. Due to being a transposon, it is also able to damage the genome, e.g. by causing insertions, deletions, etc. Furthermore, the methylation state of LINE-1 reliably represents global methylation—hence, it can be used as a biomarker of the methylation pattern [18].

Green tea polyphenols are known to exert chemopreventive effect through epigenetic pathways (e.g., regulation of gene expression, inhibition of transcription factors of inflammatory genes and oncogenes, etc.) [3, 19]. Moreover, the epigallocatechin-3-gallate (EGCG) content of green tea (*C*. *sinensis*) is able to inhibit the hypermethylation caused by DNMT1 enzyme on tumor suppressor genes [20]. However, the potential protective effects of green tea catechins (GTC), resveratrol (*trans*-3,5,4’-trihydroxystilbene), and coffee on the LINE-1 DNA methylation pattern have not yet been revealed in a chemical carcinogenesis model.

In our present in vivo animal study, we examined the DNA methylation pattern of LINE-1 in the liver, spleen and kidney of DMBA pretreated mice. One group received green tea (*C*. *sinensis*) extract (catechin content 80%), one group chinese bayberry (*Morella rubra*) extract (myricetin (3,5,7,3’,4’,5’-hexahydroxyflavone) content: 80%), and one group a flavonoid extract (with 4 grams / 100 ml added resveratrol) and one group coffee (*C*. *arabica*) extract. The results were compared to data from a DMBA-treated positive and an untreated negative control group. (For details of the experimental arrangement and applied compounds see Table 1).

The objective of the experiment was to determine how these carcinogenic/chemopreventive effects are reflected in LINE-1 DNA methylation patterns, to what extent the studied substances are able to prevent the DMBA-caused methylation changes and whether or not these can be used as potential biomarkers of that effects.

**Table 1:** Arrangement of experimental groups and treatments.

		experimental material				
group	ip. DMBA	daily dose /animal	producer	product and main components	latin / scientific names	quantity
**Negativ control**	-					
**DMBA control**	+					
**DMBA** **+** **flavonoid extract**	+	30 mg	Slimbios Ltd.	FruitCafe^TM^		
** **				common grape vine seed, peel	*Vitis vinifera ’Cabernet Sauvignon’*	20 g /100 ml scald
** **				erithritol	(2R,3S)-Butane-1,2,3,4-tetrol	12 g /100 ml scald
** **				resveratrol	*trans*-3,5,4’-Trihydroxystilbenetrans-3,5,4’-trihydroxystilbene	4 g /100 ml scald
** **				blackberry ’thornfree’ seed, peel	*Rubus fruticosus „Thornfree”*	2 g /100 ml scald
** **				blackcurrant seed, peel	*Ribes nigrum*	2 g /100 ml scald
** **				total polyphenol		4000–5000 mg /100 ml scald
**DMBA** **+** **green tea extract**	+	4 mg	Xi’an Longze Biotechnology Co. Ltd.	green tea	*Camellia sinensis*	
** **				total polyphenol		98.53%
** **				total catechins		80.42%
** **				EGCG	Epigallocatechin-3-gallate	50.45%
** **				coffeine	1,3,7-Trimethylxanthine	0.28%
**DMBA** **+** **coffee extract**	+	30 mg	Xi’an Longze Biotechnology Co. Ltd.		*Coffee arabica*	
** **				chlorogenic acid	3-Caffeoylquinic acid	5.03%
** **				coffeine	1,3,7-Trimethylxanthine	1.21%
**DMBA** **+** **chinese bayberry extract**	+	2.5 mg	Xi’an Longze Biotechnology Co. Ltd.	chinese bayberry	*Morella rubra*	
** **				myricetin	3,5,7,3’,4’,5’-Hexahydroxyflavone	80.42%

## Materials and methods

In our experiment, we used six groups of 12-week-old female CBA/Ca mice (n = 6). The untreated and DMBA-treated control groups did not receive pre-feeding, while one group received 4 mg/day/animal green tea (*C*. *sinensis*) extract (Xi’an Longze Biotechnology Co. Ltd.), one group 2,5 mg/day/animal chinese bayberry (*M*. *rubra*) extract (Xi’an Longze Biotechnology Co. Ltd.) and one group 30 mg/day/animal a flavonoid extract (common grape vine (*Vitis vinifera ’Cabernet Sauvignon’*) seed and peel, blackberry ’thornfree (*Rubus fruticosus „Thornfree”*) seed and peel, blackcurrant (*Ribes nigrum*) seed and peel with added resveratrol 4 grams in 100 ml), namely FruitCafe^TM^ (Slimbios Ltd.) and one group received (30 mg/day/animal of 150 ml) coffee (*C*. *arabica*) extract for two weeks, in addition to their usual diet. With the exception of the untreated control group, the other five groups received 20 mg/bwkg DMBA intraperitoneally (Sigma-Aldrich), dissolved in 0.1 ml corn oil. After 24 hours of DMBA exposure, animals were sacrificed by cervical dislocation and then their liver, kidneys, and spleen were removed. (Table 1).

Mice were housed according to the animal experimentation principles and guidelines. All efforts were made to minimize suffering. The experiment was conducted in compliance with the current ethical regulations (Ethical license no.: BA02/2000-79/2017 was permitted by Animal Welfare Committee of University of Pécs).

### Isolation of DNA

DNA isolation was performed with High Pure PCR Template Preparation Kit (Roche, Madison, WI, USA), according to the manufacturer’s instructions.

### LINE-1 DNA methylation

EpiTect Bisulfite kit (Qiagen, Hilden, Germany) was used for bisulfate-conversion, according to the instructions provided by the manufacturer. This process resulted in the conversion of unmethylated cytosines into uracil. Subsequently, a high-resolution melting analysis (HRM) was performed, which, based on the melting point difference, was able to distinguish between uracil and methylated cytosine bases. If the DNA contains highly methylated regions, the bisulfite conversion and subsequent amplification results in a higher melting point, because retaining more cytosines leads to a higher GC content of the amplified fragment (there are 3 hydrogen bonds between guanine and cytosine). In case of less methylated regions the unmethylated cytosines are converted into adenine, resulting in a lower melting temperature.

For the HRM analysis primers targeting a CpG rich region of LINE-1 were used [21], the sequences were as follows: forward: 5’-GGT TGA GGT AGT ATT TTG TGT G-3’, reverse: 5’- TCC AAA AAC TAT CAA ATT CTC TAA C-3’. The amplification was performed in 96 well plates, in a Roche LightCycler480 qPCR instrument (Roche, Madison, WI, USA). The reaction mix contained 20 ng bisulfite treated DNA, 0.75–0.75 μM forward and reverse primers, 1x LightCycler 480 High Resolution Melting Master (Roche, Madison, WI, USA) in 20 μl final volume [21]. The PCR parameters were as follows: heating to 95°C for 5 min was followed by 35 cycles: 1. 95°C for 20 sec, 2. 60°C for 30 sec, 3. 72°C for 20 sec. Subsequently the melting point / melting curve analysis was performed between 73°C and 84°C with a temperature steps of 0.1°C/2 sec.

For the purpose of positive and negative controls Mouse high methylated genomic DNA (EpigenDx, Hopkinton, MA, USA) and Mouse low methylated genomic DNA (EpigenDx, Hopkinton, MA, USA), and their mixtures in different proportions were used, in order to allow quantification of the methylation levels of our samples.

### Calculation and statistical analysis

Relative LINE-1 methylation of expression levels were calculated and compared using the 2^-ΔΔCT^ method. During the statistical analysis for the testing the distribution of results we used the Kolmogorov-Smirnov test. To compare the averages we used the Levene’s type F-probe and T-probe. IBM SPSS 21 statistical software was used for calculations and analysis. We determined the level of statistical significance at a p value <0.05.

Average DNA methylation levels were expressed as percentages of the animals without any treatment (negative controls).

## Results

**Fig 1:**
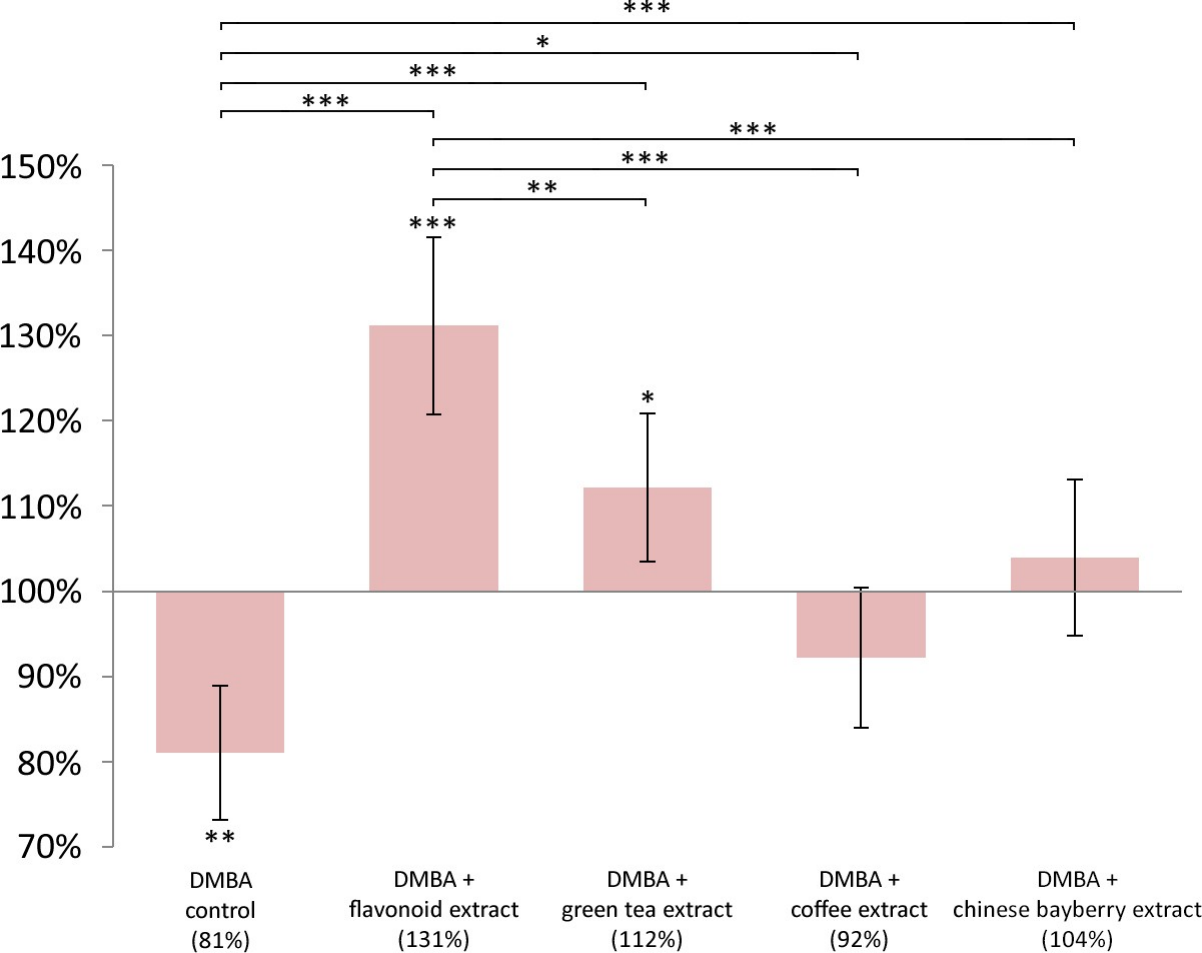
LINE -1 methylation pattern in the liver of mice. LINE-1 methylation pattern in the liver of CBA/Ca female mice (n = 6) due to the effect of DMBA, flavonoid extract, green tea extract, coffee extract and chinese bayberry extract, expressed as the ratio of untreated control (* p <0.05, ** p <0.005, *** p <0.001).

**Fig 2:**
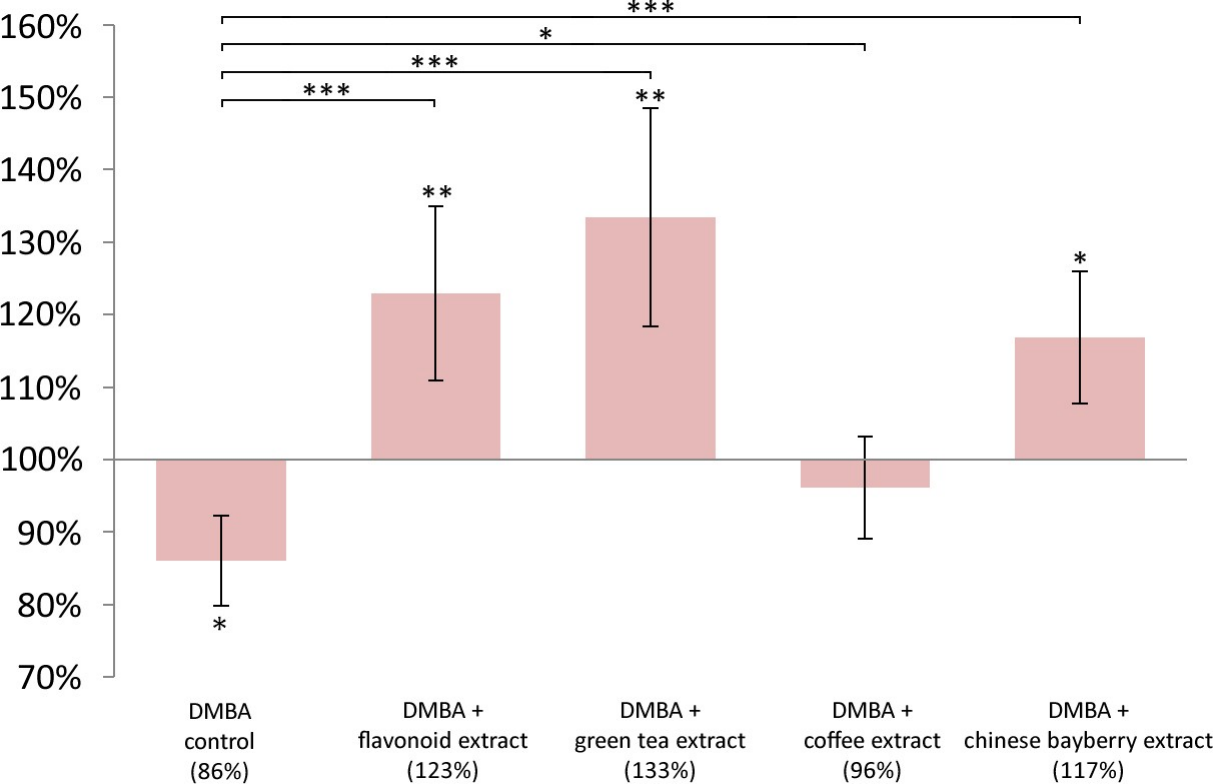
LINE -1 methylation pattern in the spleen of mice. LINE-1 methylation pattern in the spleen of CBA/Ca female mice (n = 6) due to the effect of DMBA, flavonoid extract, green tea extract, coffee extract and chinese bayberry extract, expressed as the ratio of untreated control (* p <0.05, ** p <0.005, *** p <0.001).

**Fig 3:**
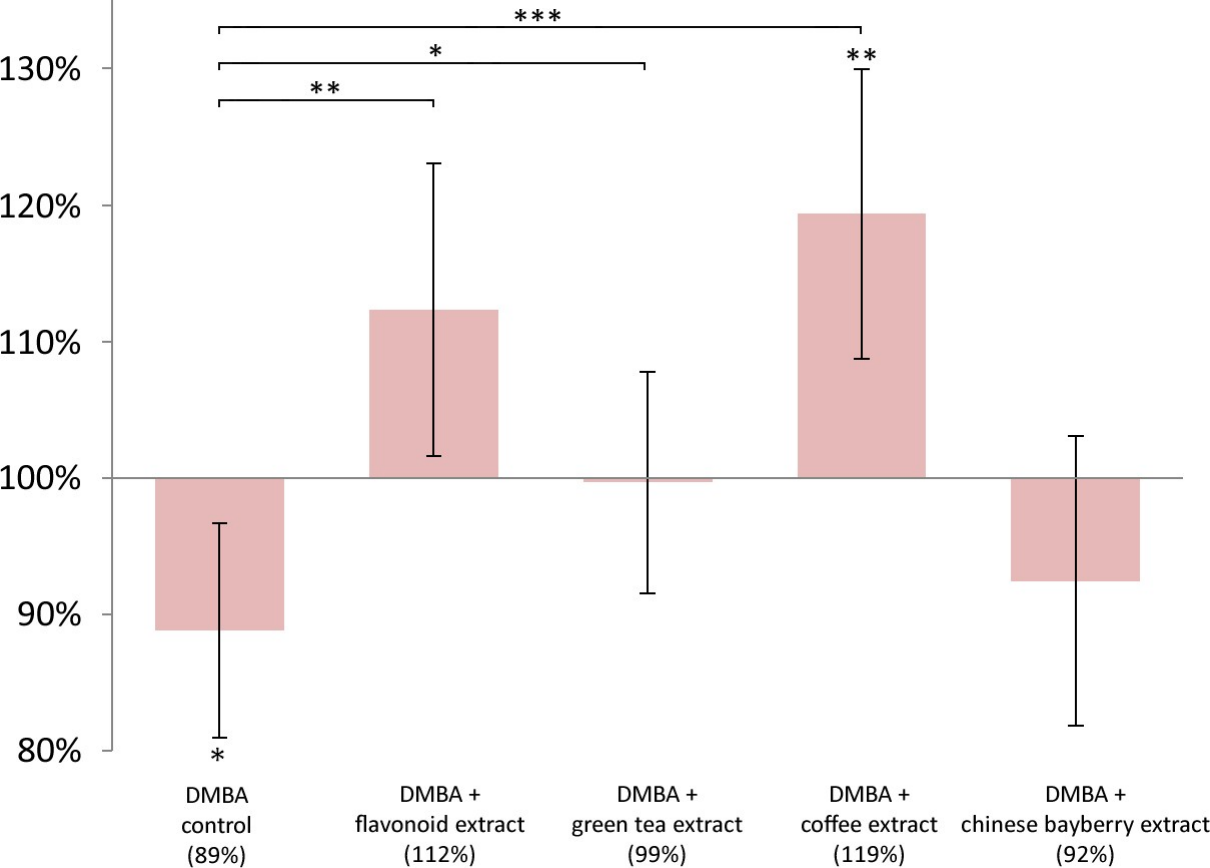
LINE -1 methylation pattern in the kidneys of mice. LINE-1 methylation pattern in the kidneys of CBA/Ca female mice (n = 6) due to the effect of DMBA, flavonoid extract, green tea extract, coffee extract and chinese bayberry extract, expressed as the ratio of untreated control (* p <0.05, ** p <0.005, *** p <0.001).

DMBA induced significant LINE-1 DNA hypomethylation in liver (p = 0.0041), spleen (p = 0.0180), and kidneys (p = 0.0444) as well, compared to controls (Figs 1–3).

The mean LINE-1 DNA methylation level compared to negative control in the liver was in DMBA treated group 81%, in coffee group 92%, in flavonoid extract group 131%, in green tea extract group 112% and in chinese bayberry group 104% (Fig 1). The mean LINE-1 DNA methylation level compared to negative control in the spleen was in DMBA treated group 86%, in coffee group 96%, in flavonoid extract group 123%, in green tea extract group 133% and in chinese bayberry group 117% (Fig 2). The mean LINE-1 DNA methylation level compared to negative control in the kidneys was in DMBA treated group 89%, in coffee group 119%, in flavonoid extract group 112%, in green tea extract group 99% and in chinese bayberry group 92% (Fig 3).

In comparison with the low LINE-1 DNA methylation level of DMBA treated group, coffee (p = 0.0377; p = 0.0259; p = 0.0002), flavonoid extract (p <0.0001; p < 0.0001; p = 0.0015), and green tea extract (p <0.0001; p <0.0001; p = 0.0401) significantly prevented the hypomethylating effect of DMBA in the mentioned organs (Figs 1–3). The chinese bayberry extract provided significant protection against this effect of DMBA only in the liver (p <0.0009) and spleen (p <0.0001) (Figs 1 and 2).

Flavonoid extract compared to untreated control (p = 0.0003), coffee (p <0.0001), green tea extract (p = 0.0065) and chinese bayberry extract (p <0.0007) also caused significant hypermethylation in the liver (Fig 1). In the same organ, green tea extract caused significant (p = 0.0456) hypermethylation compared to the untreated control (Fig 1). Compared to the untreated DMBA control, flavonoid extract (p = 0.0054), green tea (p = 0.0012) and myricetin extract (p = 0.0137) caused significant hypermethylation in the spleen, also (Fig 2). In the kidneys, the coffee consumption caused significant hypermethylation (p = 0.0068) compared to the LINE-1 DNA methylation of the negative control group (Fig 3).

## Discussion

DMBA resulted in a significant L1-RTP DNA hypomethylation compared to the methylation pattern measured in the negative control group, which, at least partly could be caused by inhibition of the DNMT enzyme [22]. Furthermore, DMBA also causes hypomethylation of oncogenes (e.g., *HA-RAS*), and hypermethylation of tumor suppressor genes (e.g., *P53*) by affecting the methylation pattern of CpG islands of DNMT enzymes themselves [23]. Consequently, these can lead to increased cell proliferation and thus increase the possibility of carcinogenesis. This is further enhanced by the fact, that DMBA–through the generation of reactive oxygen species (ROS) [24]–depletes glutathione (GSH) [24–26], and thus indirectly decreases the level of S-adenosylmethionine (SAM) and of S-adenosyl homocysteine (SAH), as well [27]. Although the decrease in SAH stimulates the DNMT1 enzyme [28], depletion of the SAM substrate ultimately supports oncogenic hypomethylation, thus promoting malignant transformation [29, 30]. Furthermore, GSH regulates cell proliferation even by increasing the expression of non-coding RNAs (ncRNA) and by affecting histone post-translational modification (HPTM) [31]. GSH is the major intracellular sulfhydryl-group donor [31]. However, injury of other participants in the methionine cycle–e.g. due to oxidative damage–, ultimately reduces the amount of GSH [31]. Thus, these feedback mechanisms also support that DMBA treatment (and other, at least partially oxidatively harmful noxas) may cause hypomethylation of L1-RTP DNA, and it is suggested that antioxidants–while protecting GSH–are also chemopreventive in this respect [30].

**Fig 4:**
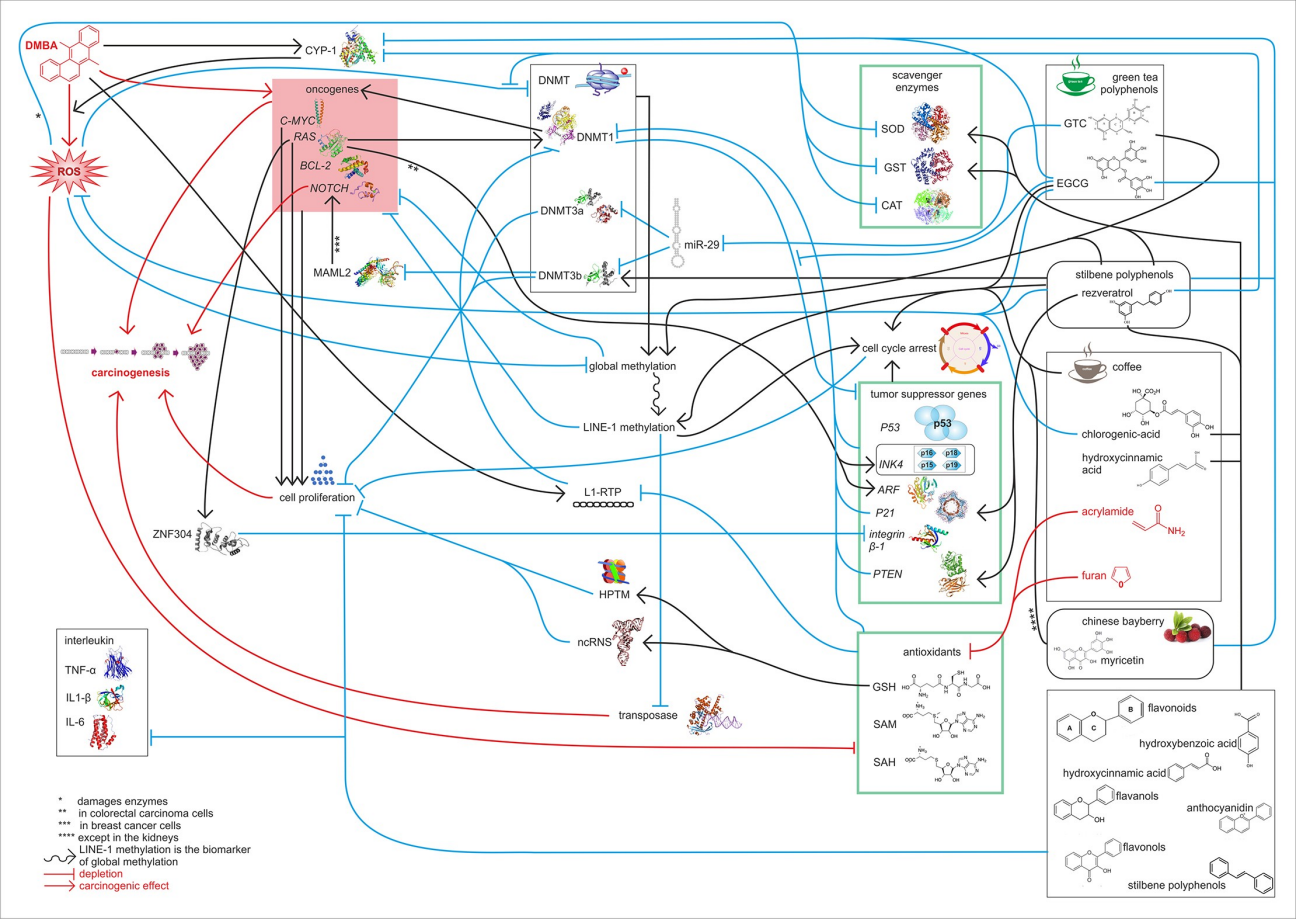
Summary of molecular mechanisms and signal transductions. The harmful effects induced signal transductions and the chemopreventive effects too influence the LINE-1 methylation pattern.

Indeed, oxidative damage may substantially contribute to global hypomethylation [32]. This can also be induced by DMBA treatment, which, besides the oncogene-activating mutations in the RAS proto-oncogene family initiates the early steps of carcinogenesis [32]. The activated K-RAS protein was demonstrated to be able to accelerate the hypermethylation of transcription factors of tumor suppressor genes (e.g. *INK4-ARF*) in several colorectal carcinoma cell lines [33], and thereby to epigenetically silence the expression of these tumor suppressor genes. Activated K-RAS also inhibits the degradation of ZNF304 transcription factor which is a transcriptional regulator of tumorsuppressor β1 integrin [34]. However, resveratrol [35], EGCG [36] and myricetin [37] can ameliorate these harmful effects by inhibiting the enzyme CYP1A1, which activates the procarcinogenic form of DMBA to an active carcinogen (while DMBA induce CYP1A1) [38]. This was confirmed by our results, where all tested substances significantly inhibited hypomethylation; moreover, hypermethylation was observed in the flavonoid consuming group compared to the untreated controls compared with other chemopreventive substances (Figs 1–3). This is explained by the fact that flavonoid consuming group ate besides the added resveratrol also the seed and peel scald of common grape vine (*V*. *Vinifera ’Cabernet Sauvignon’*), blackberry ’thornfree’ (*R*. *fruticosus „Thornfree”*) and blackcurrant (*R*. *nigrum*) (Table 1). These herbal extracts are abundant in 4-hydroxybenzoic acid, 4-hydroxycinnamic acid, flavanol, flavonol, anthocyanidin, and stilbene polyphenols, which are antioxidants, antiinflammatory (e.g. they inhibit TNF-α, IL-1β, IL-6), anticarcinogenic (e.g. they regulate cell proliferation) [39–41]. Nevertheless, the mentioned chemopreventive effects are synergistic (Fig 4).

According to literature, resveratrol prevents LINE-1 hypomethylation induced by both oxidative and inflammatory damage [42]. The chlorogenic acid (3-caffeoylquinic acid) and hydroxycinnamic acid (4-hydroxycinnamic acid) content of coffee [43], the GTC [44], and the flavonoids as well as resveratrol [45] have a scavenger effect that reduces ROS damage. Moreover, these substances also induce the antioxidant superoxide dismutase (SOD) and glutathione S-transferase (GST) enzymes [46–48], supporting the DMBA-induced hypomethylation inhibitory results of the three referred groups here (Figs 1–3). This is further supported by the fact that “GSH replacing” antioxidants, such as of alpha-tocopherol acetate, N-acetyl cysteine, methionine, SAM, and folic acid methyl group donors also reversed the phenomenon of LINE-1 hypomethylation [49]. Since oxidative stress induces hypermethylation of the repetitive LINE-1 elements of SOD enzyme family and GSTM5 promoters [50, 51] as well, and these positive feedback mechanisms have been disrupted by the substances studied in our model.

DNA methylation and histone methylation are epigenetic factors that play a central role in tumor biology and are involved in the antitumor effects of polyphenols (e.g. by resveratrol and catechins) [30, 52]. miRNAs may also play a significant role in the DNA methylation pattern, as, for example, miR-29 inhibits DNMT3a and DNMT3b enzymes in several tumor types [53]. This results in an increased rate of cell cycle, however, in line with our experimental results, this is prevented by GTC as GTC is able to reduce the expression of miR-29 [54].

The studied polyphenols also influence the methylation pattern through secondary signaling pathways, for example resveratrol increases the expression of *phosphatase and tensin homolog deleted on chromosome 10* (*PTEN*) leading to inhibition of DNMT1 [30]. Moreover, in a breast cancer cell line, the promoter of *MAML2* oncogene is demethylated, leading to the activation of *MAML2*, which activates the *NOTCH* oncogene signaling pathway [55]. However, resveratrol is able to remethylate *MAML2* via the enzyme DNMT3B, in vitro [55]. This arrests the NOTCH cascade because *MAML2* is a co-activator of NOTCH [55]. On the other hand, the regulatory role and pathological significance of the LINE-1 methylation pattern is indicated by the fact, that in patients with hypomethylated LINE-1 and with cyclin-dependent kinase-6 (CDK6) amplified esophageal squamous cell carcinoma the expression of *P21* decreased [56]. This can be related to the antitumor effect of EGCG as well, as EGCG increases the expression of *P21* tumor suppressor gene in vivo through hypomethylation of the promoter region of *P21*, irrespective of the P53 tumor suppressor-induced signaling pathway [30, 57]–and P21 inhibits DNMT1 [30].

Myricetin also induces SOD and GST [45, 58], but DMBA damages both the catalase (CAT) and SOD enzymes [59, 60], and in their absence or in case of their reduced function myricetin may act as a prooxidant [61]. Myricetin was unable to prevent LINE-1 hypomethylation in the kidneys (Fig 3), probably consistent with this, where potent metabolic CYP-1 enzymes may have activated DMBA to such an extent that myricetin could exert a prooxidant effect. (However, the activity of CYP-1A2 enzyme is also high in the liver [62] still the GSH level is also higher in that organ [63]. This explains why the myricetin treatment did not decrease the LINE-1 methylation in the liver (Fig 1)).

However, the decrease in GSH (and other carcinogenic effects) caused by trace amounts of acrylamide and furan in coffee proved to be negligible as for the results of the LINE-1 methylation pattern, since these are likely to be masked by the strong beneficial effects mentioned above (Figs 1–3) [64].

Thus, we confirmed by our experiment that DMBA exposure may also be related to the role of the ROS generator in depletion of SAM, SAH, and the antioxidant GSH, which caused LINE-1 DNA hypomethylation [49]. Moreover, based on literature, the oxidative stress-induced LINE-1 DNA hypomethylation is reversible, so probably the examined substances may exert a protective effect (at least partially) not only by pre-feeding but also during subsequent consumption [49].

## Conclusion

Our results suggest that DMBA-induced LINE-1 hypomethylation may reliably represent the prooxidant environmental effect, as well as the chemopreventive effects of the studied antioxidant compounds. Moreover, the polyphenol extract, rich in resveratrol, caused LINE-1 hypermethylation even in comparison with the untreated control group, presumably due to its effect on secondary signaling pathways. Thus, our test system is able to detect the resultant of these harmful and chemopreventive effects. This is indicated by the results observed in the kidneys of the group receiving myricetin, where the LINE-1 biomarker test system confirmed the potential prooxidant effect of myricetin associated with increased DMBA exposure [62].

Based on these, it can be assumed that the studied model can detect the effects of other oxidative harmful environmental carcinogens or of other harmful substances, as well as to demonstrate the beneficial effects of other antioxidant chemopreventive substances. In summary the LINE-1 DNA methylation pattern, examined in our present study was approved as a proper molecular epidemiological biomarker.

## Supporting information

S1 Data List of abbreviations. (XLS)

S2 Data The raw data of DMBA induced LINE-1 DNA methylation pattern, influenced by green tea extract, a Chinese bayberry extract, flavonoid extract (with added resveratrol) and coffee extract. (XLSX)
